# Diffuse Large B-Cell Lymphoma with t(1;22)(q21;q11.2) and t(6;18)(p25;q21): A Case Report

**DOI:** 10.3390/reports8010005

**Published:** 2025-01-05

**Authors:** Toshiaki Nagaie, Yasushi Kubota, Ichiro Hanamura, Sivasundaram Karnan, Rika Tomimasu, Michiaki Akashi, Shiho Tsuruda, Akiyoshi Takami, Shinya Kimura, Masaharu Miyahara

**Affiliations:** 1 Department of Internal Medicine, Karatsu Red Cross Hospital, Karatsu 847-8588, Japan; rika-tomimasu@karatsu.jrc.or.jp (R.T.); miyahara@karatsu.jrc.or.jp (M.M.); 2 Department of Clinical Laboratory Medicine, Saga-Ken Medical Centre Koseikan, Saga 840-8571, Japan; kubota-yasushi@koseikan.jp; 3 Division of Hematology, Department of Internal Medicine, Aichi Medical University School of Medicine, Nagakute 480-1195, Japan; hanamura@aichi-med-u.ac.jp (I.H.); takami-knz@umin.ac.jp (A.T.); 4 Department of Biochemistry, Aichi Medical University School of Medicine, Nagakute 480-1195, Japan; skarnan@aichi-med-u.ac.jp; 5 Department of Pathology, Karatsu Red Cross Hospital, Karatsu 847-8588, Japan; michiaki-akashi@karatsu.jrc.or.jp; 6 Department of Laboratory Medicine, Karatsu Red Cross Hospital, Karatsu 847-8588, Japan; shiho-tsuruda@karatsu.jrc.or.jp; 7 Division of Hematology, Respiratory Medicine and Oncology, Department of Internal Medicine, Faculty of Medicine, Saga University, Saga 849-8501, Japan; shkimu@cc.saga-u.ac.jp

**Keywords:** diffuse large B-cell lymphoma, fluorescence in situ hybridization, 1q21, immunoglobulin lambda, *BCL2*

## Abstract

**Background and Clinical Significance**: This should include a brief introduction about the general medical condition or relevant symptoms that will be discussed in the case report and should succinctly summarize the critical essential clinical information of the case report and emphasize its new and vital aspects. **Case Presentation**: A 72-year-old man diagnosed with DLBCL involving chromosomal translocations t(1;22)(q21;q11.2) and t(6;18)(p25;q21) showed primary refractory disease after the fourth cycle of R-CHOP. The patient ultimately experienced cardiac involvement due to the lymphoma and received salvage chemotherapy. He passed away about 15 months after the diagnosis of DLBCL. We conducted fluorescence in situ hybridization (FISH) for further analysis of the chromosomal translocations. The breakpoint of chromosome 1q21 was located at a distance of around 151 Mb from the telomeric end of chromosome 1p. The breakpoint in chromosome 22q11 contains the *immunoglobulin lambda* locus. Furthermore, the breakpoint of chromosome 6p was in the telomeric region of chromosome 6p21. The breakpoint of chromosome 18q21 contains *BCL2*. **Conclusions**: This case report presents the first documented co-occurrence of chromosomal translocations t(1;22)(q21;q11.2) and t(6;18)(p25;q21) in a patient with DLBCL. These chromosomal translocations may indicate a worse clinical outcome.

## 1. Introduction and Clinical Significance

Non-Hodgkin lymphoma (NHL) is the most common hematological malignancy, and diffuse large B-cell lymphoma (DLBCL) is the most frequent subtype of NHL, accounting for 30–40% of cases. DLBCL results from the clonal proliferation of a germinal or post-germinal malignant B cell. DLBCL is categorized into two subtypes based on the cell of origin: germinal center B-cell-like (GCB) and activated B-cell-like (ABC). In general, the ABC subtype has a poorer prognosis compared to the GCB subtype. The diagnosis is generally made from the biopsy of the suspicious lymph node. The standard frontline treatments for most patients with DLBCL remain rituximab and cyclophosphamide, doxorubicin, vincristine, and prednisone (R-CHOP), with or without radiation [1]. However, further classification of treatment for patients with DLBCL based on variants or subgroups is generally not established. The 5-year overall survival (OS) in DLBCL ranges from 79.5% for stage I to 54.7% for stage IV disease, with a median 5-year OS of 64.6%. Despite receiving standard treatment, up to 30–40% of patients with DLBCL either show disease progression during or at the end of treatment (primary refractory) or relapse after the initial response [2]. This is suspected because DLBCL is an aggressive disease with heterogeneous genetic, morphological, phenotypic, molecular, and clinical characteristics [2,3,4,5,6,7,8,9]. Therefore, characterizing primary refractory or relapse patients at the individual level may facilitate the development of effective treatment strategies aimed at improving the overall prognosis of DLBCL.

The search for chromosomal abnormalities is an important finding for the diagnosis of malignant lymphoma subtypes and prognosis estimation. For example, t(14;18)(q32;q21) is a chromosomal translocation that is characteristic of follicular lymphoma, but it is also detected in a certain proportion of DLBCL cases [10]. Here we report a novel case of DLBCL coexisting with chromosomal translocations t(1;22)(q21;q11.2) and t(6;18)(p25;q21). This case demonstrated primary refractory disease during R-CHOP treatment and exhibited an aggressive clinical course. We conducted fluorescence in situ hybridization (FISH) analysis to examine the precise chromosomal breakpoints, and, as far as our knowledge extends, no similar case has been previously reported.

## 2. Case Presentation

A 72-year-old man was referred to our hospital with a fever >38 °C, fatigue, and elevated lactate dehydrogenase (LDH). His Eastern Cooperative Oncology Group performance status (ECOG PS) was 1. He had a medical history of bladder cancer at the age of 68 years, which was treated with transurethral resection plus intravesical bacillus Calmette–Guerin therapy. The use of 18F-fluorodeoxyglucose positron emission tomography/computed tomography (18F-FDG PET/CT) for bladder cancer staging revealed increased 18F-FDG accumulation in the left submandibular lymph node with a maximum standardized uptake value (SUVmax) of 6.8, the left subclavian lymph node with an SUVmax of 4.7, the mesenteric lymph node with an SUVmax of 3.5, and the paraaortic lymph node with an SUVmax of 3.9. Because there were no superficial lymph nodes available for biopsy, he was followed up with imaging due to suspicion of low-grade NHL, which had remained stable for about 4 years. In addition, his medical history included chronic atrial fibrillation, reflux esophagitis, and a previous surgery for ossification of the ligamentum flavum. He was prescribed apixaban for atrial fibrillation and rabeprazole sodium for reflux esophagitis. He had no history of familial genetic disorder, smoking, or known allergies to food or medication.

**Figure 1:**
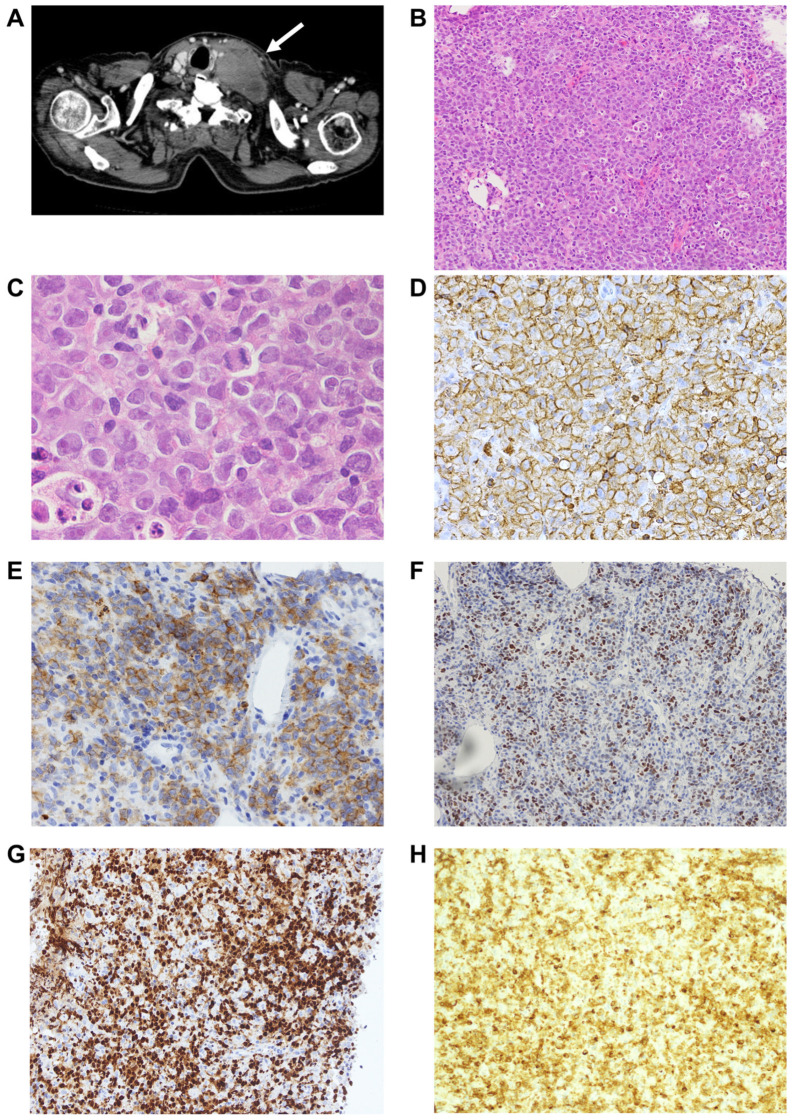
Cervical computed tomography (CT), histology of the lymph nodes and the bone marrow, and the chromosomal analysis at the time of our hospital referral. (**A**) Contrast-enhanced CT showing enlarged lymph nodes in the left neck (arrow). (**B**) Hematoxylin and eosin (H&E) staining of the lymph nodes (magnification 20×). (**C**) H&E staining of the lymph nodes (magnification 100×). (**D**–**H**) Immunostaining of the lymph nodes with an anti-CD20 (**D**), CD10 (**E**), BCL6 (**F**), MUM1 (**G**), and BCL2 (**H**) (magnification 40×).

At the time of our hospital referral, physical examination revealed palpable enlarged lymph nodes extending from the left neck to the left submandibular region. Blood analysis showed the following findings: hemoglobin, 145 g/L; white blood cell count, 8.1 × 10^9^/L (93% segmented neutrophils, 7% lymphocytes); platelet count, 331 × 10^9^/L; LDH, 808 U/L (normal range, 124–222 U/L); C-reactive protein, 6.3 mg/L (normal range, 0.0–1.4 mg/L); and soluble interleukin-2 receptor alpha (sIL-2Rα), 2393 U/mL (normal range, 122–496 U/mL). Hepatitis B surface and core antibodies were positive, but hepatitis B virus DNA was not detected. CT imaging demonstrated a rapid increase in the size of the left cervical lymph nodes, measuring 7.5 cm × 4.9 cm, which were compressing his trachea and thyroid (Figure 1A). The mesenteric and paraaortic lymph nodes were also enlarged, but their size remained similar to previous imaging during the follow-up. A needle biopsy of the largest left cervical lymph nodes was performed, which revealed diffuse infiltration by medium- to large-sized atypical lymphocytes, which disrupted the normal lymph node structure. (Figure 1B,C). These lymphocytes were found to be positive for CD10, CD20, CD79a, MUM1, BCL2 (90%), and BCL6 (Figure 1D–H), but negative for CD3, CD5, and Epstein–Barr virus-encoded small RNA (EBER). The Ki-67 labeling index was 80%.

**Figure 2:**
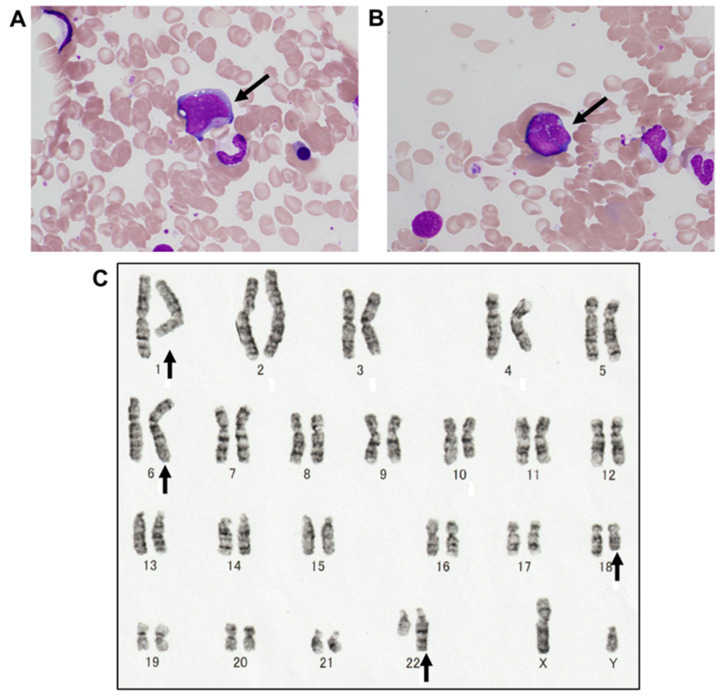
Analyses of bone marrow cells. (**A**,**B**) May–Grünwald Giemsa staining of the bone marrow (magnification 100×). The arrow indicates an abnormal lymphocyte in each image. (**C**) G-banded karyotyping showed t(1;22)(q21;q11.2) and t(6;18)(p25;q21). The arrows indicate translocated chromosomes.

Bone marrow (BM) aspiration showed 2.4% of abnormal lymphocytes that are large-sized, with vacuoles in the cytoplasm, nuclear irregularities, and prominent nucleoli (Figure 2A,B). These lymphocytes were CD20 positive, and cytogenetic abnormalities, specifically 46, XY, t(1;22)(q21;q11.2), and t(6;18)(p25;q21), were observed in 20/20 of the metaphases analyzed (Figure 2C). Flow cytometry of BM samples showed 12% lymphocytes, with T lymphocytes comprising 69%. The immunophenotype of T lymphocytes was positive for CD2, CD3, CD4, CD5, CD7, and weakly positive for CD8. The ratio of CD4/CD8 lymphocytes was 2.0. B lymphocytes, which comprised 19%, were weakly positive for CD10, CD19, and CD20. The ratio of kappa and lambda immunoglobulin light chains, determined by flow cytometry using the BM cells, was 0.2 (normal range, 0.3–3.0) [11]. The upper endoscopy showed no evidence of lymphoma invasion. The cardiac echocardiogram showed mild mitral regurgitation, moderate pulmonary hypertension, but normal left ventricular systolic function. The electrocardiogram showed atrial fibrillation.

He was diagnosed as having DLBCL, of which the cell of origin was determined to be a GCB type according to Hans’ algorithm [12]. The clinical stage was IVB. According to the National Comprehensive Cancer Network International Prognostic Index (NCCN-IPI), his NCCN-IPI score was 6, placing him in the high-risk group [13]. The patient received R-CHOP (rituximab 375 mg/m^2^ on day 1, cyclophosphamide 750 mg/m^2^ on day 2, doxorubicin 50 mg/m^2^ on day 2, vincristine 2 mg on day 2, and prednisone 100 mg on days 2–6). Oral fluconazole was administered for fungal prophylaxis, and oral trimethoprim-sulfamethoxazole was administered to prevent *Pneumocystis jirovecii* pneumonia. He experienced a grade 4 neutropenia according to the Common Terminology Criteria for Adverse Events (CTCAE). He received granulocyte colony-stimulating factor and his neutrophil count was recovered. After the third cycle of R-CHOP, CT imaging showed continued lymph node shrinkage. Additionally, the bone marrow aspiration did not detect any apparent abnormal lymphocytes and showed a normal karyotype. However, the left cervical lesion enlarged again after the fourth cycle of R-CHOP. We added radiation therapy (40 Gy/20 Fr) for the enlarged left cervical lesion. Although the tumor had re-grown after the R-CHOP four-course treatment, because it was a localized lesion, we chose to use radiation therapy instead of immediately proceeding to salvage chemotherapy. The patient received a total of six cycles of R-CHOP.

The patient visited the nearest hospital with the chief complaints of palpitations, dizziness, and loss of consciousness. He was diagnosed with sustained ventricular tachycardia there and subsequently referred to our hospital. Further analysis revealed that the right ventricle of the heart was ultimately invaded by DLBCL, as detected by cardiac echocardiography, CT, and cardiac magnetic resonance imaging. A relapse was confirmed approximately 4 months after completing R-CHOP treatment. The sustained ventricular tachycardia was relieved with landiolol hydrochloride. To address the relapse of DLBCL, he received R-GCD (rituximab 375 mg/m^2^ on day 8, gemcitabine 1000 mg/m^2^ on days 1 and 8, carboplatin AUC = 5 on day 1, and dexamethasone 16.5 mg on days 1–4). However, prior to the fourth cycle of R-GCD, blood examinations showed an increase in LDH and soluble interleukin-2 receptor alpha levels, and CT imaging revealed an increase in pericardial fluid. He was considered to have progressive disease after the third cycle of R-GCD and was switched to Pola-RB (rituximab 375 mg/m^2^ on day 1, polatuzumab vedotin 1.8 mg/m^2^ on day 2, and bendamustine 90 mg/m^2^ on day 2–3). Even after the second Pola-RB, his disease continued to progress, and the pericardial fluid remained increased by CT imaging. However, pericardiocentesis was not performed due to the absence of findings indicating cardiac tamponade, based on the consultation with the cardiologist. He subsequently received dose-adjusted EPOCH (etoposide 50 mg/m^2^ on days 1–4, vincristine 0.4 mg/m^2^ on days 1–4, doxorubicin 10 mg/m^2^ on days 1–4, cyclophosphamide 560 mg/m^2^ on day 5, and prednisone 60 mg on days 1–5). Unfortunately, he passed away on day 6 of the first EPOCH cycle, approximately 5 months after the relapse. The cause of his death was suspected to be a lethal arrhythmia due to the progression of lymphoma.

**Figure 3:**
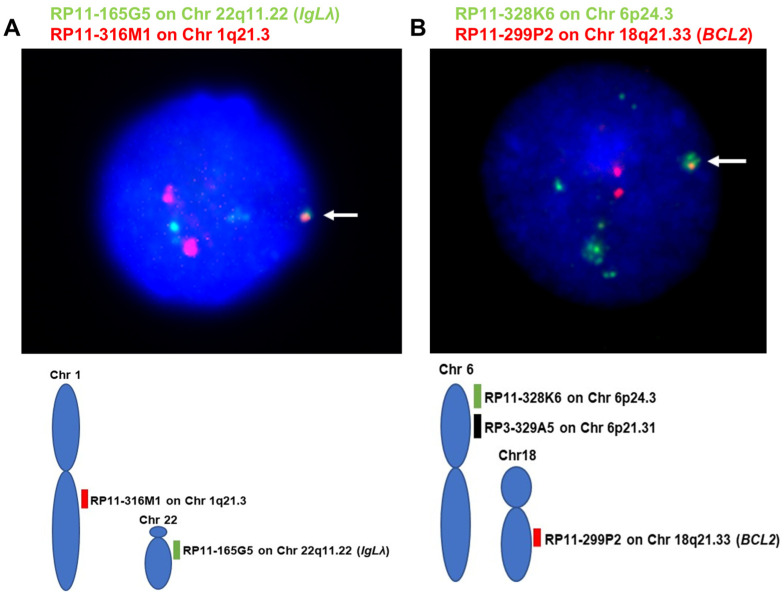
Fluorescence in situ hybridization (FISH) analysis. (**A**) FISH analysis using BAC clones RP11-316M1 (red) containing chromosome 1q21.3 and RP11-165G5 containing chromosome 22q11.22 (IgLλ, green). The arrow indicates a fusion signal of t(1;22)(q21;q11.2). (**B**) FISH analysis using BAC clones RP11-328K6 (green) harboring chromosome 6p24.3 and RP11-299P2 (red) containing chromosome 18q21.33, which contains BCL2. The arrow indicates a fusion signal of t(6;18)(p25;q21). Each lower panel is a schematic of the chromosomal location of the BAC clones.

To investigate chromosomal breakpoints of t(1;22) and t(6;18) in more detail using BM samples, FISH analysis was performed, employing bacterial artificial chromosome (BAC) clones located at chromosome bands 1q21, 22q11, 6p25, and 18q21 as probes. The analysis of t(1;22)(q21;q11.2) indicated that the breakpoints of chromosome 1q21 and 22q11 were in RP11-316M1 and RP11-165G5, respectively (Figure 3A). RP11-165G5 contains the immunoglobulin lambda (IGL) locus, suggesting that t(1;22)(q21;q11.2) may be involved in pathogenesis by regulating a certain gene located in RP11-316M1 through an IGL enhancer. The analysis of t(6;18)(p25;q21) indicated that the breakpoint of chromosome 6p was on the telomeric side of chromosome 6p24, whereas that of chromosome 18q21 was in RP11-299P2, which contains BCL2 (Figure 3B). We also performed FISH using BAC clones on the telomere side of RP11-328K6, which is located at chromosome 6p25; however, they did not hybridize well. This suggests that BCL2 may be fused with the gene on the telomeric side of chromosome 6p25 or may be under the position effect of the promoter of the gene on the telomeric side of chromosome 6p25. *MYC* (8q24) translocation was not investigated.

### 2.1. Materials and Methods

#### 2.1.1. Cytogenetic Analysis by G-Banding

Cytogenetic analysis by G-banding using the BM cells was outsourced to SRL (Tokyo, Japan). The BM cells were cultured after adding phytohemagglutinin (PHA) free culture medium and subjected to G-banding after the specimen preparation. The detailed methodology follows that of Seabright et al. [14].

#### 2.1.2. FISH

BACs located at 1q21.3 (RP11-316M1), 6p24.3 (RP11-328K6), 18q21.33 (RP11-299P2), and 22q11.2 (RP11-165G5) were purchased from BAC/PAC Resources (Oakland, CA, USA) and used for FISH probes. DNA from the BAC clones was directly labeled by nick translation with Spectrum-Green and/or Spectrum-Red according to the manufacturer’s instructions (Abbott, Abbott Park, IL, USA). Information on the BAC clones was obtained via the website of the National Center for Biotechnology Information (NCBI), and the probes were confirmed to map to the precise chromosome bands using metaphase spreads from the peripheral blood lymphocytes from healthy donors. Chromosome preparation for cytogenetics was performed according to the standard procedure. Briefly, cells were cultured in RPMI 1640 for 24 h, treated with colcemid (final concentration, 0.05 mg/mL) for 30 min, harvested with hypotonic potassium chloride (0.075M KCl), and fixed with methanol/glacial acetic acid (3:1, cornoy solution). DAPI was used for the counterstain of nuclei. The cells were examined and photographed under an Olympus BX60-RF microscope (Olympus, Tokyo, Japan). We counted 100 nuclei and considered them positive if 90 or more nuclei gave the same result.

## 3. Discussion

We present a case of chemo-refractory DLBCL who exhibited rare chromosomal translocations 46, XY, t(1;22)(q21;q11.2), t(6;18)(p25;q21). Transformation of DLBCL from low-grade NHL, like follicular lymphoma (FL), is suspected based on the clinical course. The patient did not respond to standard chemotherapy with R-CHOP, indicating primary refractory disease. Additionally, he exhibited rare cardiac involvement due to the lymphoma and experienced an aggressive clinical course, even after receiving salvage chemotherapy regimens. Regrettably, the patient passed away. FISH analysis using the BM sample was conducted. This patient was considered to have DLBCL, not otherwise specified, according to the fifth edition of the *World Health Organization Classification of Haematolymphoid Tumours: Lymphoid Neoplasms* and the International Consensus Classification of Mature Lymphoid Neoplasms [5,6].

The chromosomal abnormalities in DLBCL vary, and there is no single specific chromosomal abnormality that characterizes all cases of DLBCL. DLBCL is a heterogeneous disease with various subtypes, and there are genetic and chromosomal alterations among individual patients. Nevertheless, some characteristic genetic abnormalities have been reported in subtypes of DLBCL cases, which may impact the prognosis and treatment strategies. The chromosomal translocations that are repeatedly observed in DLBCL include the *BCL6*, *BCL2*, and *MYC* genes, and their frequencies are estimated to be 30–40%, 20–30%, and 5–15%, respectively. About 50% of *BCL6* translocations involve the immunoglobulin gene (Ig) region, but the remainder involve non-IG regions. So far, many sites have been identified as translocation partners, including 6p21 (Histon *H4*), 16p11 (*IL21-R*), 16p13 (MHC class II transactivator, *CIITA*), and 7p12 (*IKZF1*, *Ikaros*) [15,16,17]. Niitsu et al. reported that the prognosis for patients with this type of translocation alone is favorable [18]. *BCL2* (18q21) translocations cause BCL2 overexpression, and translocations of the *BCL2* and *IgH* genes are associated with GCB DLBCL, which is seen in 35% of cases. BCL2 overexpression in ABC-DLBCL is due to amplification of 18q [19]. In immunohistochemistry, BCL2 is positive in 62% of ABC DLBCL and 30% of GCB DLBCL and is a poor prognostic factor [19]. *MYC* (8q24) translocations are often t(8;14) and its variants, t(2;8) and t(8;22). In addition, overexpression due to MYC amplification has been found in 38% of DLBCL cases and is considered to be a poor prognostic factor [20]. In addition to *MYC* gene translocations, lymphomas with *BCL2* or *BCL6* translocations and lymphomas with both *MYC*, *BCL2*, and *BCL6* translocations are known as double-hit (DHL) or triple-hit (THL) lymphomas, respectively [3].

FISH analysis was performed for further analysis in our case. Frequently, 18q21 is detected in FL, and its role in the transformation from FL was highly suspected based on the FISH analysis of t(6;18)(p25;q21) [21]. In the present case, t(6;18) might cause overexpression of *BCL2* in the present case, which is associated with poor prognosis in DLBCL [22].Tamura et al. reported that FISH analysis of 173 cases of B-cell lymphoma detected *IgH* translocations in 70 cases (40.5%), and that 6p25 (*MUM1/IRF4*) was identified as a partner gene in one case [23]. Furthermore, one case of DHL showing t(14;18)(q32;q21), t(8;22)(q24;q11.2), and t(6;14;18)(p25;q32;q21) has been reported, and it is thought that there are two independent dual hit translocations of *MYC/BCL2* and *IRF4/BCL-2* [24]. In our case, MUM1/IRF4 was highly expressed, which may reflect the *MUM1/IRF4* translocation. The breakpoint of chromosome 1q21 was detected from the analysis of t(1;22)(q21;q11.2). Abnormalities of chromosome 1q21 are common in B-cell non-Hodgkin’s lymphoma, and Willis et al. identified *BCL9* involved in t(1;14)(q22;q32) [25]. In the same study, FISH analysis using an 850-kb yeast artificial chromosome spanning *BCL9* identified cases of t(1;22)(q21;q11), which causes the juxtaposition of *BCL9* to the *IGL* locus [25]. Adachi et al. reported a case of angioimmunoblastic T-cell lymphoma (AITL) with t(1;22)(q21;q11) and speculated that the detected t(1;22) was probably of B-cell origin, as there are many cases of composite lymphoma with B-cell lymphoma in AITL [26]. Because numerous genes located on chromosome 1q21 are linked to B-cell lymphoma, including *BCL9*, *MCL1*, and *IRTA1* [25,27,28,29], a gene on chromosome 1q21 in this patient might be dysregulated due to the *IGL* translocation. Consequently, the *IGL* translocation might be associated with the development and progression of lymphoma [30].

The NCCN-IPI is a valuable tool for stratifying prognostically relevant subgroups of DLBCL in the current era of rituximab-based treatment. It considers factors such as age, LDH, ECOG PS, Ann Arbor stage, and the presence of extranodal disease. Based on this assessment, the patient was classified into the high-risk group, with estimated 5-year overall survival (OS) at 33% and 5-year progression-free survival at 30% in the NCCN cohort, which implies a poor prognosis.

This patient with DLBCL, harboring chromosomal translocations 46, XY, t(1;22)(q21;q11.2), t(6;18)(p25;q21), exhibited primary refractory disease to R-CHOP. Subsequently, salvage chemotherapy was administered at the timing of the relapse after completing 6 cycles of R-CHOP. Unfortunately, he passed away approximately 15 months after being diagnosed with DLBCL. Notably, the NCCN-IPI does not consider chromosomal abnormalities. By incorporating chromosomal abnormalities into the assessment, we may achieve a more precise prognosis and make more effective treatment strategy decisions to improve the prognosis in patients with DLBCL.

Cardiac lymphoma is categorized into primary cardiac lymphoma and secondary cardiac lymphoma. In our case, it was classified as secondary lymphoma due to cardiac involvement observed at the time of relapse. Secondary cardiac involvement due to lymphoma is more common compared to primary cardiac lymphoma and has been reported in up to 25% of patients with nodal disease [31]. The most prevalent form of secondary cardiac involvement occurs in patients with DLBCL. In most cases of secondary cardiac lymphoma, symptoms are attributed to heart failure or rhythm alterations [32], which is similar to our case. While there are no established guidelines for the management of cardiac lymphoma, treatment approaches for cardiac lymphoma involve chemotherapy, often combined with radiotherapy, surgery, autologous hematopoietic stem cell transplantation, and chimeric antigen receptor (CAR) T-cell therapy [33,34]. Unfortunately, in our case of secondary cardiac lymphoma, autologous hematopoietic stem cell transplantation and CAR T-cell therapy were not considered due to the patient’s age and overall general condition.

We selected R-GCD, Pola-RB, and EPOCH as the salvage treatments for this patient. For the patients ineligible for transplantation or CAR T-cell therapy, polatuzumab vedotin in the Pola-RB regimen is an effective treatment in challenging-to-treat settings. It delivers monomethyl auristatin E, a microtubule inhibitor, to B-cells [35]. Regarding EPOCH, which includes doxorubicin, despite its potential for cardiac toxicity, we chose it because the anthracycline dosage did not reach the upper limit. Additionally, his ventricular tachycardia was caused by secondary cardiac lymphoma rather than cardiac toxicity.

There are also some limitations in our case report. First, a definite diagnosis was not made at the age of 68, and he was subsequently followed up as having a low-grade NHL with imaging. Considering the clinical course, this patient was suspected to have transformed from a low-grade NHL to DLBCL. Furthermore, the present case is GCB type, so there is a possibility of DHL, but MYC translocation has not been examined. Second, due to the insufficient sample volume obtained from the lymph node needle biopsy, the FISH was performed using the BM samples at the age of 72, which were used to detect chromosomal translocations. Third, we were not able to identify genes on 1q21 and 6p25 using the available analytical methods.

## 4. Conclusions

To the best of our knowledge, this is the first case report of DLBCL coexistent with chromosomal translocations t(1;22)(q21;q11.2) and t(6;18)(p25;q21). Referring to the literature, it is assumed that *BCL2*, *BCL9*, and *MUM1/IRF4* are the molecules that have been translocated. The strong expression of BCL2 and MUM1 may also support this assumption. These translocations may indicate a poor clinical prognosis. To gain a better understanding of the biological and clinical significance of these translocations, it is crucial to accumulate more cases similar to ours to improve prognosis in the future.

## Data Availability

The original contributions presented in this study are included in the article. Further inquiries can be directed to the corresponding author.
